# Development of dynamic kinetic resolution on large scale for (±)-1-phenylethylamine

**DOI:** 10.3762/bjoc.6.97

**Published:** 2010-09-13

**Authors:** Lisa K Thalén, Jan-E Bäckvall

**Affiliations:** 1 Stockholm University, Department of Organic Chemistry, Arrhenius Laboratory SE-106 91, Stockholm, Sweden

**Keywords:** dynamic kinetic resolution, kinetic resolution, racemization, Shvo, *Candida antartica* lipase B

## Abstract

*Candida antarctica* lipase B (CALB) and racemization catalyst **4** were combined in the dynamic kinetic resolution (DKR) of (±)-1-phenylethylamine (**1**). Several reaction parameters have been investigated to modify the method for application on multigram scale. A comparison of isopropyl acetate and alkyl methoxyacetates as acyl donors was carried out. It was found that lower catalyst loadings could be used to obtain (*R*)-2-methoxy-*N*-(1-phenylethyl)acetamide (**3**) in good yield and high ee when alkyl methoxyacetates were used as acyl donors compared to when isopropyl acetate was used as the acyl donor. The catalyst loading could be decreased to 1.25 mol % Ru-catalyst **4** and 10 mg CALB per mmol **1** when alkyl methoxyacetates were used as the acyl donor.

## Introduction

Chiral amines are important building blocks in the synthesis of many pharmaceuticals, fragrances, and agricultural products, and it is therefore important to develop methods for their preparation that are applicable on multigram scale. They can be prepared by resolution of amines, hydrogenation of prochiral imines and enamines [1–2], alkylation of prochiral imines [3], aminohydroxylation of alkenes [4], transamination of prochiral ketones [5–7], and reductive amination of prochiral ketones [8]. Of these methods kinetic resolution using enzymes is often favored due to its simplicity [9–10]. The main disadvantages of kinetic resolution are that only a maximum yield of 50% can be achieved and that the remaining unreacted starting material must be removed from the product mixture. However, by racemizing the slower reacting enantiomer in situ, in a process known as dynamic kinetic resolution (DKR) [11], a theoretical yield of 100% can be achieved.

**Scheme 1:**
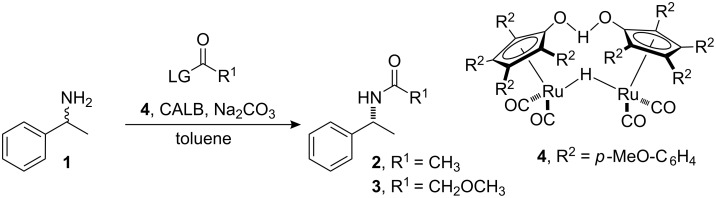
Dynamic kinetic resolution of (*rac*)-1-phenylethylamine.

We have previously developed a highly efficient protocol for the DKR of primary amines using *Candida antarctica* lipase B (CALB) as the enzyme and **4** as the racemization catalyst (See Scheme 1 for an example of the previously developed protocol with isopropyl acetate as the acyl donor providing **2** as the amide product) [12–13]. The Jacobs–De Vos group showed that palladium on an alkaline earth support in combination with an enzyme can be used for practical DKR of benzylic amines [14–16], while the Kim and Park group have combined a palladium nanocatalyst with an enzyme for the DKR of amines [17]. Additional procedures for the chemoenzymatic DKR of amines in which various racemization methods are combined with enzymatic resolution have been established [18–23]. We herein report the evaluation of our system for application on multigram scale.

## Results and Discussion

**Figure 1:**
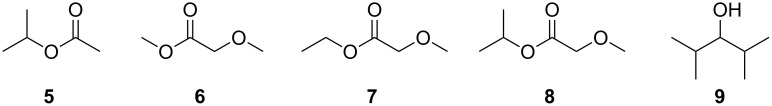
Acyl donors and hydrogen donor used in DKR.

The previously reported [12–13] protocol for application of DKR on a 0.5 mmol scale was evaluated with the aim of developing a system that could be utilized on a larger scale (10–50 mmol). The previously reported conditions for application of DKR to **1** (Scheme 1) were as follows: 4 mol % racemization catalyst **4**, 20 mg CALB, 6.8 equivalents isopropyl acetate (**5**) (Figure 1), and 20 mg sodium carbonate in 8 mL of toluene at 90 °C (Table 1, entry 1). These conditions were used as a starting point and the reaction parameters were changed individually and then concurrently in a continuing attempt to find the ideal conditions for a large scale reaction. The reaction parameters evaluated included catalyst loading, concentration of the reaction, and the quantity of acyl donor used.

**Table 1:** Investigation of individual DKR parameters.^a^

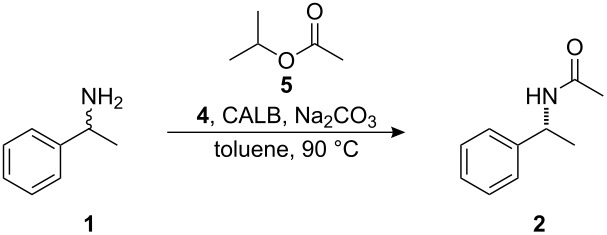					

Entry	**4** (mol %)	**5** (equiv)	Conc (M)	ee (%)	Yield^b^ (%)

1	4	6.8	0.06	98	90
2	4	3.4	0.06	98	67
3	4	1.5	0.06	99	51
4	3	6.8	0.06	98	84
5	2	6.8	0.06	98	81
6	1	6.8	0.06	97	72
7	4	6.8	0.13^c^	98	93

^a^Conditions: 0.50 mmol of **1**, Ru-catalyst **4**, 20 mg of CALB, 20 mg of Na_2_CO_3_, isopropyl acetate (**5**), 8 mL of toluene, 90 °C, 72 h; ^b^determined by GC analysis; ^c^4 mL of toluene.

Changes of individual reaction parameters were examined first. It was found that a marked decrease in the reaction rate was observed when the excess of acyl donor **5** was decreased from 6.8 equiv to 1.5 equiv (Table 1, entries 1–3). It was possible to decrease the Ru-catalyst loading to 1 mol % and still obtain **2** in 72% yield with 97% ee (Table 1, entry 6). The reaction was then concentrated to see if there was an effect on the rate of the reaction and on byproduct formation. It was found that the reaction was slightly accelerated and only small amounts of byproduct were observed when the reaction mixture was concentrated from 0.06 M to 0.13 M (Table 1, entries 1 and 7, respectively).

The parameters were then changed concurrently to find a balance between the rate of acylation and racemization at lower catalyst loadings. When the Ru-catalyst loading was decreased to 2 mol % and the reaction concentration was increased to 0.25 M, the amide **2** could be obtained in 77% yield with 99% ee (Table 2, entry 1). When the Ru-catalyst loading was further decreased to 1 mol%, and the amount of CALB or acyl donor was increased, a decrease in the enantioselectivity of the reaction was observed (Table 2, entries 2 and 3, respectively) indicating that the racemization was slower than the transesterification at these catalyst loadings. An increase in the selectivity was observed when the amount of acyl donor was decreased, however this also coincided with deceleration of the reaction rate (Table 2, entries 4–6). The concentration of the reaction was increased to 2.5 M, resulting in a faster yet less selective reaction (Table 2, entry 7). In a final attempt to obtain **2** in high yield and ee, the Ru-catalyst loading was again increased to 2 mol %. Amide **2** was obtained in 64% isolated yield with 97% ee (Table 2, entry 8). Further attempts to find appropriate conditions at high concentrations (1–3 M) resulted in significant byproduct formation.

**Table 2:** Investigation of concurrent changes of DKR parameters with **5** as acyl donor.^a^

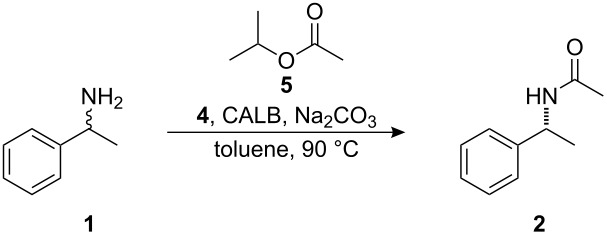							

Entry	**1** (mmol)	**4** (mol %)	**5** (equiv)	Conc (M)	CALB/Na_2_CO_3_ (mg/mmol **1**)	ee (%)	Yield^b^ (%)

1	0.5	2	6.8	0.25	40/40	99	77
2	2.5	1	7	0.25	80/60	90	86 (73)
3	5	1	10	0.25	40/40	89	76 (72)
4^c^	1	1	3	0.20	10/20	98	53
5	2.5	1	1.5	0.25	40/60	>99	40
6	2.5	1	1.15	0.50	40/60	99	55^d^
7	5	1	3	2.5	40/40	93	(61)^e^
8	10	2	1.5	2.9	30/30	97	(64)^f^

^a^Conditions: 40 mg of CALB per mmol **1**, Ru-catalyst **4**, isopropyl acetate (**5**), toluene, 90 °C, 72 h; ^b^determined by GC, isolated yields in paranthesis; ^c^2.5 mmol of hydrogen donor **9** (Figure 1); ^d^18% byproduct formation; ^e^6% byproduct formation; ^f^21% byproduct formation.

Since it proved difficult to obtain both a high enantiomeric excess value and a high yield at a reduced catalytic loading, and that the amount of acyl donor used played a key role in the outcome of the results, another acyl donor was tested.

A 200-fold acceleration in the kinetic resolution of **1** has been observed with methyl methoxyacetate **6** (Figure 1) as the acyl donor instead of methyl butyrate [24]. However, it is also known that an uncatalyzed chemical acylation of the substrate occurs at elevated temperatures when using acyl donors of this type in the DKR of primary amines. This has been overcome by initially adding one equivalent of the acyl donor followed by a later addition of 0.1 equiv of the acyl donor to allow the reaction to go to completion [22].

The addition of 1.5 equiv of ethyl methoxyacetate **7** (Figure 1) in one portion in the application of DKR to **1** with 1 mol % **4** and 20 mg CALB at 90 °C was tested. As expected, the product amide **3** was obtained with a low ee (83%) after 72 hours (Table 3, entry 1). The consecutive addition of **7** in different ratios at 0, 24, and 48 hours was then tested. The amount of hydrogen donor **9** (Figure 1), CALB, sodium carbonate and the concentration of the reaction was varied concurrently. Alcohol **9** was added as a hydrogen donor to suppress byproduct formation [25]. Some general trends could be observed. The concentration of the reaction had a large impact on the rate of uncatalyzed acylation. This could be seen in the decrease of enantiomeric excess observed when going from a concentration of 0.2 M to 0.5 M (Table 3, entries 2 and 4). Also, a decrease in the amount of enzyme to compensate for the decrease in loading of racemization catalyst gave both a better enantiomeric excess value and a better yield (Table 3, entries 2–3). Addition of 1 equiv acyl donor **7**, followed by addition of 0.5 equiv after 24 hours led to unsatisfactory ee values (Table 3, entry 2–4). By only adding 0.1 equiv **7** after 24 hours, an enantiomeric excess of 97% could be obtained (Table 3, entry 5).

**Table 3:** Investigation of concurrent changes of DKR parameters with **7** as acyl donor.^a^

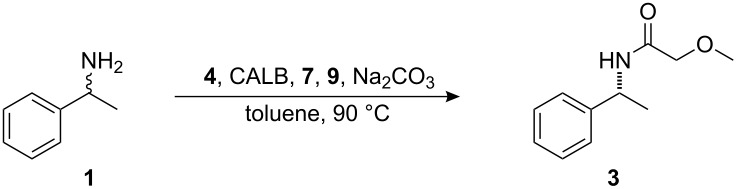							

Entry	Ethyl methoxyacetate (**7**) (equiv)			Hydrogen donor **9** (mmol)	CALB/Na_2_CO_3_ (mg)	ee (%)	Yield (%)^b^

	0 h	24 h	48 h				

1	1.5	—	—	2.5	20/40	83	99
2	1	0.5	—	2.5	20/40	86	79
3	1	0.5	—	2.5	10/20	90	97
4^d^	1	0.5	—	1	20/40	74	88
5^c^	1	0.1	—	1	20/40	97	87
6^c^	1	—	0.2	1	20/40	96	83
7	1	0.15	—	1.25	10/20	95	70
8^e^	1	0.15	—	2.5	10/20	97	91
9	0.75	0.35	—	2.5	10/20	94	73

^a^Conditions: 1 mmol of **1**, 1 mol % of **4**, 10 mg of CALB, 20 mg of Na_2_CO_3_, ethyl methoxyacetate (**7**), 2.5 mmol of hydrogen donor **9**, 5 mL of toluene, 90 °C, 72 h; ^b^determined by GC; ^c^10 mL of toluene; ^d^2 mL of toluene; ^e^100 °C.

The substrate amine was no longer racemic after 24 hours, indicating that the racemization was the slower of the two reactions. In order to allow the racemization more time, acyl donor **7** was added after 48 hours instead of after 24 hours; however, this did not improve the overall outcome of the reaction (Table 3, entry 6). The addition of 1 equiv **7** was then compared to the addition of 0.75 equiv **7** at the beginning of the reaction. A sample was taken after 24 hours and enantiomeric excesses of 97% and 99%, respectively were obtained for **3**. Subsequently, 0.15 equiv and 0.35 equiv **7**, respectively were added. After a reaction time of 72 hours, **3** was obtained in 95% and 94% ee, respectively (Table 3, entry 7 and 9). The higher ee observed after 24 h indicates that a smaller portionwise addition was advantageous. Furthermore, when the reaction temperature was increased to 100 °C both the enantiomeric excess and the yield improved (Table 3, entry 8).

Additionally, methoxyacetate type acyl donors **6**–**8**, which differ in the leaving group, were investigated. When acyl donor **6** was used in the DKR of **1**, amide **3** was obtained with the highest ee (97%) and in 89% yield (Table 4, entry 1). However, when acyl donor **8** was used in the DKR of **1**, amide **3** was obtained with a lower ee (88%) but in higher yield (97%, Table 4, entry 3). When **7** was employed as acyl donor, **3** was obtained in 91% yield with an ee of 95%. Thus, an increase in the enantiomeric excess of **3** corresponded with a decrease in the size of the leaving group (i.e. **6** > **7** > **8**), and an increase in the yield corresponded with an increase in the size of the leaving group (i.e. **6** < **7** < **8**, Table 4, entries 1–3).

**Table 4:** Acyl donor screening.^a^

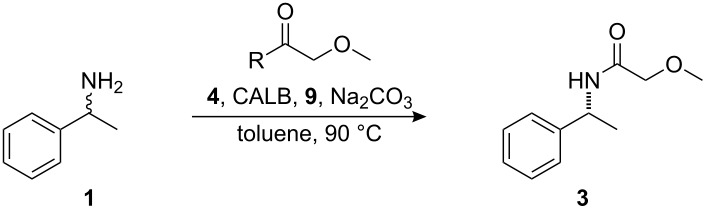					

Entry	Acyl donor	R	Time (h)	ee (%)	Yield^b^ (%)

1	**6**	OMe	24	99	70
			72	97	89
2	**7**	OEt	24	98	73
			72	95	96
3	**8**	O-*i*Pr	24	94	74
			72	88	97

^a^Conditions: 1 mmol of **1**, 1 mol % of **4**, 10 mg of CALB, 20 mg of Na_2_CO_3_, 1 mmol of acyl donor (0.15 mmol added after 24 h), 2.5 mmol of hydrogen donor **9**, 5 mL of toluene, 90 °C, 72 h; ^b^determined by GC.

Acyl donors **6** and **7** were both evaluated in the DKR of **1** on 10 mmol scale. First, a two portion addition of the acyl donor in 0.75 equiv and 0.35 equiv aliquots was tested. Under these conditions, use of acyl donor **6**, provided amide **3** with a slightly higher ee than with the corresponding addition of acyl donor **7** (Table 5, entries 1 and 3). Also, a three portion addition of acyl donor **7** provided the product **3** with a higher ee than from a two portion addition (Table 5, entries 1–2). Since a slightly higher ee was obtained with acyl donor **6**, it was used in the DKR of **1** on 45 mmol scale. When using a three portion addition of acyl donor **6** and a three portion addition of CALB, amide **3** was obtained with 98% ee and in 68% isolated yield (Table 5, entry 4). A two portion addition of acyl donor **6** and CALB provided **3** in 83% isolated yield with an ee of 98% (Table 5, entry 5).

**Table 5:** Scale up of dynamic kinetic resolution.^a^

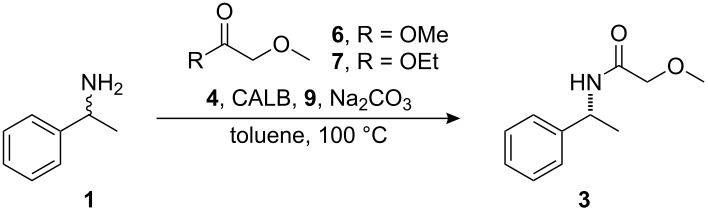								

Entry	**1** (mmol)	Acyl donor	Acyl donor (equiv)			**8** (equiv)	ee (%)	Yield (%)^b^

			0 h	24 h	48 h			

1	10	**7**	0.75	0.35	—	2.5	97	93 (90)
2	10	**7**	0.4	0.35	0.35	2.5	99	91
3	10	**6**	0.75	0.35	—	1.25	98	(74)
4^c^	45	**6**	0.4	0.35	0.35	1.25	98	(68)
5^d^	45	**6**	0.75	0.35	—	1.25	98	(83)

^a^Conditions: **1**, 1.25 mol % of **4**, 100 mg of CALB, 200 mg of Na_2_CO_3_, acyl donor, hydrogen donor **9**, 50 mL of toluene, 100 °C, 72 h; ^b^determined by GC, isolated yield in parenthesis; ^c^225 mg of CALB, addition of 113 mg of CALB after 24 and 48 h; ^d^340 mg of CALB, addition of 110 mg of CALB after 24 h.

## Conclusion

The application of DKR to (±)-1-phenylethylamine (**1**) has been investigated for use on multigram scale. A comparison of isopropyl acetate and alkyl methoxyacetates as the acyl donor was carried out. It was found that lower catalyst loadings could be used to obtain (*R*)-2-methoxy-*N*-(1-phenylethyl)acetamide (**3**) in good yield and with high ee when alkyl methoxyacetates were used as the acyl donor than when isopropyl acetate was used as the acyl donor. The catalyst loading could be decreased to 1.25 mol % Ru-catalyst **4** and 10 mg CALB per mmol **1** when alkyl methoxyacetates were used as the acyl donor. Application of DKR with these catalyst loadings and either ethyl methoxyacetate (**7**) or methyl methoxyacetate (**6**) as the acyl donor provided (*R*)-2-methoxy-*N*-(1-phenylethyl)acetamide (**3**) in 90% yield and with 97% ee (10 mmol scale) and 83% yield with 98% ee (45 mmol scale), respectively.

## Experimental

### General

Unless otherwise noted, all manipulations were performed under an argon atmosphere. Toluene was dried with a VAC solvent purifier. Flash chromatography was carried out on 60 Å (35–70 µm) silica gel. ^1^H and ^13^C NMR spectra were recorded at 400 MHz and at 100 MHz, respectively. Chemical shifts (δ) are reported in ppm, using the residual solvent peaks in CDCl_3_ (δ_H_ 7.26 and δ_C_ 77.00) as internal standards, and coupling constants (*J*) are given in Hz. The enantiomeric excess was determined by analytical GC employing a CP-Chirasil-DEX CB column (25 m Ø × 0.32 mm). Racemic compounds were used as references.

#### Dynamic Kinetic Resolution

**45 mmol scale:** (*R*)-2-methoxy*-N*-(1-phenylethyl)acetamide, **3**. A flame dried 1 L two-necked round-bottomed flask was charged with Novozym 435 (340 mg), Na_2_CO_3_ (900 mg, 8.5 mmol), and Ru-complex **4** (745 mg, 0.56 mmol). The vessel was evacuated and backfilled with argon three times. Toluene (225 mL), (±)-1-phenylethylamine (**1**, 5.8 mL, 45 mmol), 2,4-dimethyl-3-pentanol (8 mL, 57 mmol), and methyl methoxyacetate (3.4 mL, 34 mmol) were added subsequently via syringe. The mixture was stirred at 100 °C. After 24 h the reaction vessel was allowed to cool for 10 min at rt. Methyl methoxyacetate (15.8 mmol, 1.6 mL) and Novozym 435 (110 mg) were added. The reaction mixture was then stirred at 100 °C. After a reaction time of 72 h, the reaction was cooled to rt and the solids were removed by filtration through a sintered glass funnel. The solvent and other volatiles were subsequently removed in vacuo. Crystallization from Et_2_O:pentane afforded (*R*)-2-methoxy-*N*-(1-phenylethyl)acetamide (**3**) as an off-white powder (5.62 g, 65%, >99% ee). The mother liquid was subsequently purified by kugelrohr distillation to afford a further quantity of (**3**) (1.60 g, 18%, 93% ee). The combined yield from crystallization and distillation was 83% (7.22 g, 98% ee). ^1^H NMR (400 MHz, CDCl_3_) δ = 7.38–7.30 (m, 4H), 7.30–7.24 (m, 1H), 6.74 (br s, 1H), 5.22–5.14 (m, 1H), 3.99 (d, J = 15.0, 1H), 3.87 (d, J = 15.0, 1H), 3.41 (s, 3H), 1.51 (d, J = 6.9, 3H). ^13^C NMR (101 MHz, CDCl_3_) δ = 168.7, 143.1, 128.8, 127.5, 126.3, 72.1, 59.3, 48.1, 22.0. Chiral GC-analysis (CP-Chirasil-DEX CB column (25 m Ø × 0.32 mm)): Injector 250 °C Program: 100 °C / 5 min / 155 °C / 3 °C min^−1^, 5 min / 200°C / 20 °C min^−1^, 5 min. *t*_S_= 20.44 min, *t*_R_= 21.00 min. [α]^27^_D_ = + 91.3 (c = 1.0, CDCl_3_).

**10 mmol scale:** (*R*)-2-methoxy-*N*-(1-phenylethyl)acetamide, **3**. A flame dried 250 mL Schlenk tube was charged with Novozym 435 (100 mg), Na_2_CO_3_ (200 mg), and Ru-complex **4** (166 mg, 0.125 mmol). The vessel was evacuated and backfilled with argon three times. Toluene (50 mL), (±)-1-phenylethylamine (**1**, 1.3 mL, 10 mmol), 2,4-dimethyl-3-pentanol (3.5 mL, 25 mmol), and ethyl methoxyacetate (1.6 mL, 7.5 mmol) were added subsequently via syringe. The mixture was stirred at 100 °C. After 24 h the reaction vessel was allowed to cool for 10 min at rt. Ethyl methoxyacetate (3.5 mmol, 410 μL) was added. The reaction mixture was then stirred at 100 °C. After a reaction time of 72 h, the reaction was cooled to rt and the solids were removed by filtration through a sintered glass funnel. The solvent and other volatiles were subsequently removed in vacuo. The crude product was purified by column chromatography (DCM:MeOH:NH_3_, 98:2:0.1 to 90:10:0.1) to afford 1.73 g (90%) of (*R*)-2-methoxy-*N*-(1-phenylethyl)acetamide (**3**) in 97% ee.
